# Domestication Reduces Floral Volatile Richness in Squash (Cucurbitaceae: *Cucurbita*) But Conserves Key Compounds Critical for Pollinator Attraction

**DOI:** 10.1007/s10886-025-01664-5

**Published:** 2025-12-03

**Authors:** Avehi Singh, Swayamjit Ray, Kristen K. Brochu-DeLuca, Andrew J. Myrick, Nathaniel B. McCartney, Jared G. Ali, Margarita M. López-Uribe

**Affiliations:** 1 Department of Entomology, The Pennsylvania State University, University Park, PA 16802 USA; 2 Intercollege Graduate Degree Program in Ecology, The Pennsylvania State University, University Park, PA 16802 USA; 3 Department of Biology, University of Arkansas at Little Rock, Little Rock, AR USA; 4 Department of Biology, University of The Bahamas, Nassau, New Providence Bahamas

**Keywords:** Domestication, Pollinator, Olfaction, Floral volatile, Squash

## Abstract

The domestication of crops leads to profound changes on plant phenotypes, yet its effects on floral traits mediating plant-pollinator interactions remain poorly understood. Floral volatile organic compounds (VOCs) play a crucial role in pollinator attraction by signaling the presence and quality of available floral resources. Here, we characterize VOC composition in *Cucurbita*, a genus containing multiple wild and domesticated species, to investigate how domestication affects floral volatiles. Then, we combined electrophysiology, blue vane trap field assays and pollinator visitation experiments to assess how the squash specialist pollinator, *Xenoglossa pruinosa*, responded to existing VOCs across different wild and domesticated plant species. Our results reveal significant compound losses within domesticated species blends. Combined gas chromatography-electroantennography (GC-EAG) identified ten electrophysiologically active compounds across wild and domesticated squash VOCs. Field assays assessing bee attraction to individual antennally active compounds using modified blue vane traps with compound lures identified 1,4-dimethoxybenzene (a dominant volatile in domesticated squash blends) as an attractant for bees in isolation. We also found significant associations between bee visitation (assessed as floral approaches and nectaring behaviors) and increased emissions of 1,4-dimethoxybenzene, dihydro-β-ionone, and (*E*)-nerolidol as well as reduced emissions of linalool and methyl salicylate. Our findings provide novel insights into the chemical ecology of crop-pollinator interactions, demonstrating that domestication can reshape plant-pollinator communication mechanisms.

**Supplementary Information:**

The online version contains supplementary material available at 10.1007/s10886-025-01664-5.

## Introduction

In the Anthropocene, the agriculturalization of landscapes is a dominant phenomenon with approximately 40% of terrestrial land mass being occupied by crop plants that have been domesticated throughout the past 10,000 years (Ellis et al. 2010). Domestication involves breeding plants for human-beneficial traits (e.g., larger fruit and higher sugar content) or traits adaptive to agroecosystems (e.g., loss of dormancy and shifts in annual life cycle) (Fuller et al. 2023). This process of artificial selection leads to rapid evolution that results in large phenotypic changes over a short period of time. Due to multiple, complex genetic processes such as pleiotropy, epistasis, linkage, and environmental factors, artificial selection often has unintended effects on plant phenotypes (Singh and van der Knaap 2022), including changes in traits involved in communication with other organisms (Chen et al. 2018; Geslin et al. 2017). For instance, domesticated plants often look, smell, and taste different from related wild species, all of which can impact how they are perceived by insects (Glasser et al. 2023; Whitehead et al. 2017). Flowers function as lures to attract pollinators by producing cues that may advertise the availability of nutritious resources (mainly pollen and nectar), mating sites, or even emulate potential mates (Burger et al. 2010; Chittka and Raine 2006; Raguso 2004). Floral cues are diverse and include visual, olfactory, humidity, and electromagnetic signals that pollinators often detect by integrating inputs from multiple sensory systems (Burger et al. 2010; Chittka and Raine 2006; Nordström et al. 2017; Dahake et al. 2025). Disruption of floral cue signals due to crop domestication can have profound impacts on plant-pollinator interactions (Brochu-DeLuca et al. *in review*) and pollinator evolution (Pope et al. 2023). However, our knowledge of how crop domestication impacts floral cues and the communication channels between plants and their pollinators is limited.

Flowers produce a complex blend of volatile organic compounds (hereafter, VOCs) that allow foraging insects to locate them over larger spatial scales than through visual cues (Schiestl 2015). Many insect pollinators possess highly sensitive olfactory systems tuned to detect small amounts of these floral volatile cues (Murray et al. 2024; Schiestl 2015). Further, floral VOC blends also vary across individual plants and may reflect underlying stress that they may be experiencing, acting as honest cues of the quality of the resources offered by a particular plant, helping the pollinator to hone in on high-quality resources while foraging (Hambäck 2016; Knauer et al. 2021; Kooi et al. 2023). There is evidence that domestication alters plant volatile profiles in cotton, but these changes do not impact herbivore preference (Hagenbucher et al. 2016). Although comparisons of the chemical composition of wild and domesticated blueberry nectar and pollen showed that domestication had significantly reduced chemical diversity, potentially impacting the health of visiting bumble bees (Egan et al. 2018), less is known about the ways in which domestication affects floral volatile profiles.

The most important and common group of insect pollinators of crops are bees, a clade of obligate pollinivores whose evolutionary history is closely intertwined with that of flowering plant species (Almeida et al. 2023; Danforth et al. 2006). Bee pollinators include both generalists—that collect pollen from a spectrum of plant families—and specialists—that collect pollen from a limited set of plant species (Waser and Ollerton 2006). The importance of floral volatiles to generalist and specialist foraging bees has been well established. For instance, generalist honey bees (*Apis mellifera*) learn odor cues associated with specific pollen sources (Arenas and Farina 2012; Reinhard et al. 2010) and the specialist *Andrena vaga* is highly sensitive to specific behavior-modulating odorants produced by host flowers (Burger et al. 2013). Generalist and specialist bees may use olfactory cues differently, as foraging involves unique challenges for each. Specialist bees tend to use specific blends emitted by their host plants to accurately locate flowers in heterogeneous environments (Burger et al. 2013, 2021). In contrast, generalists tend to show sensory biases towards volatile compounds present across floral blends (Dötterl and Vereecken 2010; Haber et al. 2021), but see (Heuel et al., 2024)).

The chemical ecology of specialized plant-pollinator interactions is a rapidly advancing field of study. Specialist bees have been shown to be particularly dependent on olfactory cues during foraging, using volatiles to locate host plants over long-distances (Friberg et al. 2014; Riffell 2011). In multiple systems, specialist pollinators show sensory biases for the dominant volatile compounds or blends emitted by their host plants (Burger et al. 2021; Carvalho et al. 2014; Milet-Pinheiro et al. 2013). For instance, *Andrena vaga*, a species that specializes on pollen from the plant genus *Salix*, uses 4-oxoisophorone, a floral VOC produced by many *Salix* species, to accurately detect the plants it needs (Burger et al. 2013). Further explorations of the neurophysiology and neuroecology of this species have shown that the peripheral olfactory system is sensitive toward this compound (Burger et al. 2021; Polidori et al. 2020). For all these systems, electrophysiological approaches have been particularly useful in identifying individual compounds and blends that may drive insect behavior and sensory evolution (Struble and Arn 1984). These approaches allow us to directly identify individual compounds detected by the peripheral nervous system and test their behavioral relevance, thus linking species ecology with underlying neurophysiological mechanisms.

Here, we use the plant genus *Cucurbita* and its associated specialist pollinators as a model to explore how plant domestication has impacted floral detection and behavior by specialist pollinators. This genus, native to North and South America, contains all the cultivated gourds and squash species and their wild ancestors (Castellanos-Morales et al. 2018; Kates et al. 2017). The first domestication event of *Cucurbita* occurred approximately 10,000 years ago in Mexico, and an additional five domestication events took place throughout the Americas with subsequent cultivation of these species worldwide where these plants did not previously grow (Smith 2006). The phylogeny and biogeography of *Cucurbita* is well-resolved and wild species occupy both xeric and mesic habitats in the Americas (Kates et al. 2017). The mesophytic clade contains five monophyletic lineages, each comprising either only extant domesticated species or domesticated and wild species (Castellanos-Morales et al. 2018). The xerophytic clade consists of six wild perennial species only found in dry regions of North America within the USA and Mexico.

The pollinator community of domesticated *Cucurbita* species in what is now the Northeastern United States and Southeastern Canada is dominated by the specialist pollinator species, *Xenoglossa pruinosa,* and the generalists *Bombus impatiens* and *Apis mellifera* (Artz and Nault 2011; McGrady et al. 2020; Willis Chan and Raine 2021). Both generalist pollinators are social and often introduced in the system as managed pollinators. In contrast, the wild bee *X. pruinosa* is a solitary, univoltine bee that is an obligate pollinivore on *Cucurbita* pollen. *Xenoglossa pruinosa* originated in central Mexico and has experienced a large, recent range expansion concurrent with the domestication and widespread cultivation of squash in North America (López-Uribe et al. 2016; Pope et al. 2023). Previous studies have shown that these bees are attracted to squash floral volatiles that are also attractive to the specialist herbivore *Acalymma vittatum* (Andrews et al. 2007). However, whether domesticated and wild *Cucurbita* species use similar compounds to attract these specialist pollinators remains unknown.

In this study, we investigated the effect of domestication on the volatile profiles of wild and domesticated *Cucurbita* flowers, how these chemical compounds were detected by specialist pollinators, and how they were associated with their attraction to traps and flowers. Specifically, we used (1) GC–MS to characterize floral volatiles, (2) electrophysiological techniques to identify EAG-active compounds, and (3) behavioral assays (field attraction assays to traps and floral pollinator visitation experiments) to infer the behavioral relevance of identified compounds. We hypothesized that domestication impacts pollinator behavior in regard to squash species mediated by changes in floral volatile profiles. Specifically, we predicted that domestication leads to reductions in the floral VOC richness of *Cucurbita* species resulting in the losses of floral volatile compounds and fewer compounds detected by bee antennae. Additionally, we predicted that bee approaches and nectaring behaviors would correlate with emissions of EAG-active compounds, with stronger associations to VOCs compounds emitted by wild squash species that the bees coevolved with. We found that domesticated and wild plants produced significantly (*p* < 0.05) differing floral VOC blends, with domesticates showing a marked reduction in the richness of compounds produced. Using combined gas-chromatography electroantennography (GC-EAG), we identified ten compounds across the floral volatile blends of five species of *Cucurbita* that elicited EAG responses and found that xerophytic and mesophytic squash produced different EAG-active compounds. However, only one compound, 1,4-dimethoxybenzene, elicited squash bee attraction to traps when used in the field in isolation. Finally, experiments quantifying bee visitation to squash flowers showed that emissions of several EAG-active compounds were associated with both pollinator approaches to flowers and nectaring behavior to flowers. Our results provide new insights into the chemical ecology of squash pollination and the impact of host-plant domestication on specialist pollinator behavior.

## Materials and Methods

### Squash Floral Volatile Characterization

We sampled floral headspace volatiles from five *Cucurbita* lineages representing a wild species, two independently domesticated lineages and wild relatives: *C. foetidissima* (wild xeric species), *C. maxima* ssp. *maxima* (domesticated mesic species), *C. maxima* ssp. *andreana* (wild mesic species), *C. pepo* ssp. *ovifera* var. *texana* (feral mesic species), and *C. pepo* ssp. *ovifera* var. *ovifera* (domesticated mesic species). Individual plants for each species listed above were grown from seed in greenhouses at the Pennsylvania State University from November 2017 to April 2018. Seeds were soaked in water overnight with continuous aeration, wrapped in wet paper towels, and transferred to an incubator at 24 °C under 16 h light and 8 h dark cycle, until the emergence of a hypocotyl. They were then transferred to four-gallon pots with Metro-Mix 830 (Sun Gro Horticulture, Agawam, MA, USA) and kept in the greenhouse at 28 °C using a 16:8 h. light: dark cycle. Plants were fertilized with two tablespoons of slow-release Osmocote 18–5–8 (Scotts Company, Marysville, OH, USA) every two weeks.

Floral headspace volatiles were sampled by bagging male flowers in 12 × 15 cm oven bags (Reynolds, Lake Forest, IL, USA) that were baked at 90 °C for two hours prior to use. Bags were secured at the base of the flower using zip ties. Floral volatiles were collected for six hours from 06:00–12:00 using a flow rate of 250 ml/minute onto 30 mg of HayeSepQ (Sigma Aldrich, USA) of mesh size 80/100 mesh adsorbent in a 4 mm inner diameter Teflon and glass tube, secured with stainless steel wire mesh (Theis et al. 2009). Passive incoming air was purified using activated charcoal. We also collected a bag control during every day of sampling in order to ascertain contamination from the bag or ambient air. Volatiles were eluted in 150 µl dichloromethane (Sigma Aldrich, St. Louis, MO, USA) and 400 ng of nonyl acetate was added as an internal standard.

Samples were analyzed in an Agilent 7890 gas chromatograph with an attached 5977 mass spectrometer using positive EI at + 70 eV. Separation was achieved on a HP-5MS UI column (30 m × 0.25 mm × 0.25 µm; Agilent, Santa Clara, CA, USA). The inlet was held at 250 °C with helium as the carrier gas at a constant flow rate of 0.8 mL/minute. The instrument was operated in full-scan mode with a scan rate of 5.1 scans/second from m/z 30 to m/z 300. The ion source temperature was 250 °C and the transfer line was held at 280 °C. For each run, 1ul of sample was injected in splitless mode, the oven temperature was kept at 40 °C for 2 min and then ramped by 10 °C per minute until it reached a final temperature of 300 °C. Compounds were identified using the NIST17 and Adams mass spectral libraries in combination with published retention indices and, when available, authentic standards. Relative concentrations of each of the identified compounds were estimated by comparing the total ion chromatogram (TIC) peak areas to those of the nonyl acetate internal standard (Table S1). We emphasize that these are approximate concentrations, as we did not use calibration curves to quantify the concentrations of compounds emitted.

### Combined Gas-Chromatography Electroantennography (GC-EAG)

Additional floral volatile samples were collected for GC-EAG assays using a similar protocol as outlined above, with a few differences. Plants for all five species of *Cucurbita* except *C. foetidissima* were grown from seed as stated previously at the Penn State Campus greenhouses. We collected volatiles from *C. foetidissima* plants growing at the University of Virginia Agricultural Research Center greenhouses in Blandy, VA, USA. Floral headspace volatiles were sampled from nine male flowers per species (three flowers sampled from three individual plants) between May to July from 2021–2023 (Table S2). Headspace sampling was conducted for one hour between 06:00 and 09:00 h using the protocol described above. We also collected a bag control with an empty bag each day. Volatiles were eluted in 150 μl of dichloromethane (Sigma Aldrich, St. Louis, MO, USA) and the samples were analyzed using the GC–MS methodology outlined above with the same instruments and columns. Compound identification was performed using the NIST17 library using Chemstation (Agilent, Santa Clara, CA, USA) (Table S3). We did not add a standard to these samples and thus did not quantify emissions of volatiles.

We hand-collected male and female *X. pruinosa* bees from squash fields of mixed cultivars of pumpkin (*Cucurbita pepo*) and butternut squash (*Cucurbita moschata*) at the Russell E. Larson Agricultural Research Station in Rock Springs, PA, USA in July and August of 2021 to 2023 during their peak activity hours of 06:00 to 09:00. Bees were kept on ice until the initiation of experiments. Recordings were made using the head of a single insect; we removed the head of the insect, used stenotomy scissors to remove the distal flagellomere, and inserted a silver electrode into the base of the head. The prepared head was mounted in a custom antennal holder such that the head electrode and antennal tip were inserted into wells filled with Ringer’s solution connected to chlorided silver electrodes. The holder ensured that the antenna was centered in the airstream and the preparation was covered to prevent noise due to mechanostimulation of the antenna.

For electroantennography, we used a modified GC-EAG setup developed by Myrick and Baker (2018). Briefly, a Silflow Deans switch (Trajan, Victoria, Australia) was used to alternate the effluent between the flame ionization detector (FID) and electroantennogram (EAG) in an Agilent 6190 N GC-FID instrument. The Dean’s switch was operated externally using nitrogen gas at a frequency of 1.5 Hz. The air-flow rate through the odor delivery tube was held at 300 ml/minute. We used a 30 m Agilent DB-5 column with 0.32 mm diameter and 0.25 µm coating. We injected 1 µl of VOC extract per trial in spitless mode using the same method used with the GC–MS assays. We made recordings for five male and female squash bees for each plant volatile extract (Table S4).

To identify peaks within GC-FID traces that corresponded to compounds in our GC–MS traces, we also ran 1 µl of a saturated alkane series standard from C_7_-C_30_ in both the GC-FID instrument used for the GC-EAG runs and GC–MS instrument used for headspace characterizations. We then calculated retention times of GC-EAG peaks, relative to GC–MS values, by overlaying alkane traces across machines (Table S5). We identified compounds eliciting antennal responses on GC-FID traces (hereafter referred to as EAG-active compounds) by comparing observed retention times, retention indices, and electron ionization spectrum matches to custom libraries of retention indices of plant-associated volatile compounds, referring to previously published datasets and by comparing them to authentic standards when available (Table 1).

**Table 1:** Compounds across wild and domesticated *Cucurbita* species eliciting antennal depolarization in male and female squash bees. Compounds were identified using retention times (RT), non-polar linear retention indices (RI), electron ionization spectra and comparisons to previously generated data deposited in the NIST17 mass spectroscopy library. Compounds marked with an asterix (*) were identified using standards run on the GC–MS instrument used for volatile characterization. The table indicates the chemical class, retention time (RT) for the GC–MS traces, retention index (RI), and CAS Registry number for each identified compound

Compound	Class	RT (min)	RI	CAS
linalool^*^	Monoterpenoid alcohol	13.3	1093.33	000078–70-6
1,4-dimethoxybenzene^*^	Benzenoid	14.4	1146.18	000150–78-7
methyl salicylate^*^	Benzenoid	14.9	1202.9	119–36-8
indole	Benzenoid	16.3	1264.35	120–72-9
dihydro-β-ionone	Apocarotenoid	18.1	1450.2	17283–81-7
β-ionone^*^	Apocarotenoid	18.7	1336.56	79–77-6
(*E*)-nerolidol	Sesquiterpene	19.4	1557.68	040716–66-3

### Blue Vane Trap Field Assays

We tested for bee attraction to EAG-active compounds by using blue vane traps (BanfieldBio Inc., Seattle, WA, USA) modified to contain compound lures. We attached 2 mL plastic collection tubes onto the vanes of each trap to serve as holders for the compound lures, which consisted of open 0.8 mL tubes containing a piece of filter paper. Each compound lure was impregnated with a mixture of 20 µl of compound with 100 µl acetone or 120 µl acetone as a control. For treatments containing 1,4-dimethoxybenzene and indole, which are both solid at room temperature, we used 20 mg of each compound dissolved in 120ul acetone. These compound concentrations were validated in a previous study that used a similar trap-based method to assess the attractiveness of floral volatiles on squash bees (Andrews et al. 2007). We distributed traps within a one-acre field of mixed cultivars of pumpkin (*Cucurbita pepo*) and butternut squash (*Cucurbita moschata*) located in the Russell E. Larson Agricultural Research Station in Rock Springs, PA, USA (40°42′56″ N, 77°56′10″ W). Eight traps (seven with compound lures and one control) were placed in an equal 3 × 3 grid within the field. The traps were suspended on metal stakes using wire such that they were approximately the same height as the flowers and filled with a 10% solution of unscented Dawn Soap (Procter and Gamble, Cincinnati, OH) in water. The traps were set out from August 2nd to 14th, 2024 and checked daily for the most predominant bee species in these crops: *X. pruinosa*, *B. impatiens*, and *A. mellifera*. After checking the traps for bees at approximately 18:00 h daily, we emptied and refilled them with soap solution and fresh lures were randomly re-distributed across traps – allowing us to sample bees through the next 24-h period (Table S6).

### Pollinator Visitation Experiment

We set up an experiment to understand whether squash bee visitation was correlated with the emission of EAG-active compounds identified in this study. We used a subset of a comprehensive dataset on bee behavior across domesticated and wild squash species produced for a separate study (Brochu-DeLuca et al. *in review*) to assess correlations between squash bee behavior and normalized emissions of EAG-active compounds identified using GC-EAG. To generate the behavioral dataset, we planted eight species of domesticated and wild cucurbits (*C. foetidissima, C. maxima* ssp. *maxima, C. maxima* ssp. *andreana, C. pepo* ssp. *ovifera* var. *ovifera, C. pepo* ssp. *ovifera* var. *texana, C. argyrosperma* ssp. *argyrosperma,* and *C. argyrosperma* ssp. *sororia*) within two field plots at the Russell E. Larson Agricultural Research Station in Rock Springs, PA in 2019 and 2020 following a randomized block design. Plants within each field were divided into blocks of five individuals each, with each field having three blocks for each species. Pollinator visitation to each plant species was assessed for two weeks between July and August 2019–2020 with ten minute observation intervals repeated over 12 and 16 days. For each block, we counted instances of three behavioral events: (1) approaches (bee flying over the flower but not entering), (2) nectaring (bee landing on flower and drinking nectar), and 3) pollen collection (bee landing on flower and collecting pollen onto her back legs). Approaches and nectaring were recorded for male and female bees, while pollen collection is a behavior exclusive of females. We randomized the order in which we collected behavioral observations for each field daily. Normalized values of approaches, nectaring and pollen collection for male and female bees per plant species were generated by factoring the number of instances of each behavior by the number of open flowers per block and averaging these values across blocks (*N* = 525 observations for each species) (See Table S7). We only included visitation data for the five squash species used for GC-EAG assays in downstream analyses.

### Statistical Analyses

All statistical analyses were conducted in R version 4.4.2 (R Core Team (2024)). First, we compared compound richness across wild and domesticated species using a Kruskal–Wallis test. We visualized the distribution of volatile data across individuals in our dataset using an ordination approach and then used PERMANOVA to understand whether the composition of floral volatile profiles differed across plant species and domestication status. Given the nonlinear distributions of compound emissions and high zero-inflation within the dataset, we visualized the floral volatile profiles of samples across species using non-metric dimensional scaling (NMDS) ordination using a Bray–Curtis distance matrix. We generated the NMDS ordination using the function *‘metaMDS’* in the vegan v.2.6–8 (Oksanen et al. 2025) package in R (v.4.4.2; R Core Team (2024)). The ordination was run with 100 random starts to ensure a stable solution, and the stress value (< 0.2) was used to ensure that the model fit the data well. We assessed homoscedasticity between species and across domesticated and wild species using the *‘betadisper*’ function in vegan v.2.6–8. We then used PERMANOVA as implemented by the ‘*adonis2*’ function in vegan v.2.6–8 with 9999 permutations to test whether floral volatile composition differed across plant species and with domestication status by comparing the Bray–Curtis distances between centroids.

To test whether the number of squash bees caught in blue vane traps differed across EAG-active compounds tested, we compared summed counts of male and female squash bees caught within traps across each compound. To do this, we fit negative binomial generalized linear mixed models using glmmTMB v.1.1.11 (Brooks et al. 2017) using the sampling date as a random effect to control for temporal variation in the numbers of bees sampled. We fit the models using the BFGS optimization algorithm run with 10,000 iterations to ensure convergence and conducted pairwise comparisons between bee capture in traps baited with compounds and the control treatment using Tukey tests implemented in the multcomp v1.4.28 package (Hothorn et al. 2008).

To test whether pollinator visitation behavior was associated with the emissions of EAG-active compounds in a field context, we sought to identify linear relationships between the incidences of bee visitation, nectaring and pollen collection behaviors for each of the *Cucurbita* species used in this study with mean emissions (in mean ng/flower) of EAG-active compounds across species. We excluded the compound β-ionone from these analyses as it was only present in a single plant species within our dataset (*Cucurbita foetidissima*) and thus restricted our analyses to the emissions of six compounds. Because of the multivariate nature of our data, we assessed whether there was multicollinearity between mean compound emissions across plant species by calculating pairwise Pearson’s correlations between variables using the function ‘*cor*’ in the stats package and the variance inflation factor between variables using the *‘vif’* function in the package car v.3.1–3 (Fox and Weisberg 2019). In order to incorporate this multicollinearity into our models, we generated a representative principal component eigenvector describing variation across all three variables using a principal component analysis (PCA) implemented in the *‘prcomp’* function in package stats.

For each behavioral category (i.e., approaches, nectaring, and pollen collection), we fit a series of negative binomial generalized linear mixed models to understand whether behavioral events varied across plant species and with mean emissions (in mean ng/flower) of EAG-active compounds, substituting values for 1,4-dimethoxybenzene, linalool, and methyl salicylate with the PC1 eigenvector. We used the ‘*glmmTMB*’ function in the glmmTMB package v.1.1.10 to fit each set of models. We tested whether numbers of approaches, nectaring and pollen collection behaviors varied across plant species, setting year of data collection as a random effect. We generated pairwise comparisons across species using pairwise post hoc Tukey tests as implemented in the multcomp v1.4.28 package (Hothorn et al. 2008). We then identified relationships between squash bee behavior and EAG-active compound emissions using a second set of models, setting plant species nested within year of data collection as random effects. The negative binomial distribution was chosen to account for overdispersion in the behavior data for all models. We evaluated the significance of fixed effects using Wald tests and assessed the overall model fit using the Akaike Information Criterion (AIC).

## Results

### Effect of Domestication on Floral Volatiles

**Fig. 1:**
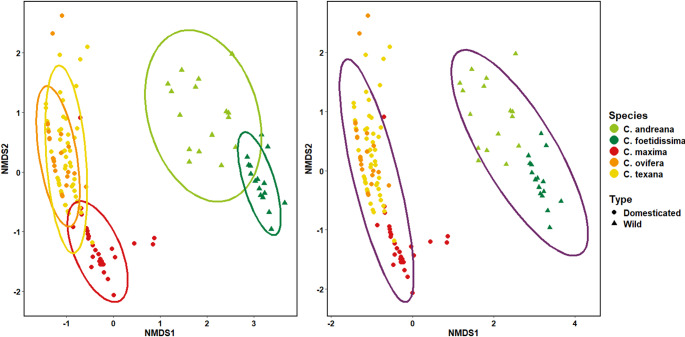
Non-metric multidimensional scaling (NMDS) ordination of floral volatile profiles across plant species used in this study. Points represent individual flowers sampled and colors represent species, as indicated in the legend. Domestication status of species is indicated by shapes (circles represent domesticated species and triangles represent wild species). Here, we classify the feral species C. pepo ssp. ovifera var. texana as domesticated given its recent divergence from the cultivated squash species C. pepo ssp. ovifera var. ovifera. (a) PERMANOVA analysis indicated significant clustering by species with dashed ellipses representing 95% confidence intervals for each species. Colors of ellipses correspond to those of plant species. (b) PERMANOVA analyses also indicated significant clustering by domestication status with dashed ellipses representing 95% confidence intervals between wild and domesticated species.

We found that domesticated plants had significantly lower floral VOC richness than wild plants (Kruskal–Wallis Χ^2^ = 109.6, df = 4, *p* < 0.001). We found that species identity explained 56% of the variation in floral volatile composition, indicating distinct volatile composition for each *Cucurbita* lineage (PERMANOVA F_4,126_ = 40.471, *p* = 0.001, R^2^ = 0.562, 999 permutations; Fig. 1). Similar analysis comparing volatiles across wild (*C. foetidissima, C. maxima ssp. andreana,* and *C. pepo* ssp. *ovifera* var. *texana*) and domesticated species (*C. maxima ssp. maxima* and *C. pepo* ssp. *ovifera* var. *ovifera*) showed significant clustering with domestication status explaining 9% of the variation (PERMANOVA F_4,129_ = 13.984, *p* = 0.001, R^2^ = 0.0978, 999 permutations), indicating that the effect size of domestication status in volatile composition is low. However, when we included *C. pepo* ssp. *ovifera* var *texana —* a feral species that has recently diverged from the domesticated *C. pepo* ssp. *ovifera* var. *ovifera —* as a domesticated species, domestication status explained 30% of the variation in volatile blends (PERMANOVA F_4,129_ = 55.207, *p* = 0.001, R^2^ = 0.300, 999 permutations; Fig. 1). We found that the dispersion of VOC profiles was significantly heterogeneous across species (F_4,126_ = 5.8897, *p* = 0.001) and between domesticated and wild species (with *C. pepo* ssp. *ovifera* var *texana* coded as a domesticated species) (F_1,129_ = 11.247, *p* = 0.002). We thus note that our results should be interpreted with caution as PERMANOVA tests are sensitive to homoscedasticity. Visual inspection of the ordination plots indicated significant overlap across species but separation across domesticated and wild categories (Fig. 1).

### Identification of EAG-active Compounds

**Fig. 2:**
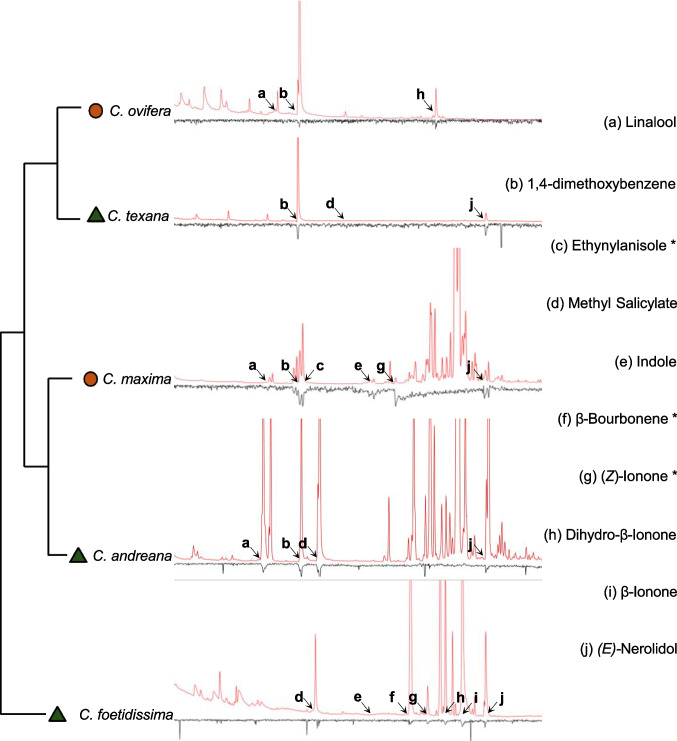
GC-EAG results for squash bees. Representative traces showing representative coupled gas chromatography-electroantennography (GC-EAG) traces for squash bees for wild and domesticated *Cucurbita* species. Outputs from the flame ionization detector (FID) (in pA) are indicated in red and outputs from the electroantennogram indicating voltage over the antenna (in mV) are indicated in black. Relationships between plant species sampled are represented by the phylogenetic tree and wild and domesticated species are indicated by green triangles and orange circles, respectively. Letters and arrows indicate FID peaks that elicited EAG responses in male and female squash bees. Compounds marked with asterisks indicate compounds that we were not able to conclusively identify and these were excluded from downstream behavioral assays

We identified ten compounds eliciting antennal responses in *X. pruinosa* bees across the five tested *Cucurbita* floral VOC blends using the GC-EAG assay (Fig. 2). Male and female bees both responded to all compounds identified, with no variation in the number of active compounds across individual bees (Fig. 2 and Table S5). We were able to identify seven out of ten compounds using previously published retention indices and available compound standards. We note that the identities of compounds for which we did not have standard information are as yet putative (Table 1). Notably, all species within the mesic *Cucurbita* clade produced the compound 1–4-dimethoxybenzene, which was absent in the wild xeric species *C. foetidissima*. This compound consistently elicited strong antennal responses in both male and female bees and was the major component of the VOC profiles of the heavily domesticated *C. pepo* ssp. *ovifera* var. *ovifera* (> 90%). 

The three compounds we were unable to confirm were not included in downstream trap assays and pollinator visitation experiments (indicated by astrices in Fig. 2). The first of these was tentatively assigned as β-bourbonene based on the partial electron ionization spectrum of the peak, but the quality of the match when compared to the NIST17 library was low. Since we did not have a standard for this compound, we tested whether bees would respond to β-bourbonene by running separate GC-EAG assays with peppermint oil, which is known to contain a trace amounts of this compound (Wu et al. 2019), but did not find any EAG active compounds in the peppermint oil. The second unidentified peak had a near match to the electron ionization spectrum of (*E)*-beta-ionone but the retention time (RT) was lower than the published RT values for that compound, hence we tentatively identified it as (*Z)*-beta-ionone. However, since neither the RT nor the calculated RI matched values for this compound, we could not be sure of the identity of this peak. The third peak was assigned as 1-ethenyl-4-methoxybenzene based on the electron ionization spectra of the peaks. However, the RT of this peak also did not conform with previously published data for this compound, and we thus concluded that the match quality was low.

### Squash Bee Attraction to Compounds in Blue Vane Trap Field Assays

We found significant variation in the number of *X. pruinosa* bees caught across compounds compared to the control treatment (Estimate = –3.60 ± 1.46, *z* = –2.47, *p* = 0.0136; Table 2). The model coefficient for 1,4-dimethoxybenzene indicated marginally higher bee capture in traps baited with this compound compared to the control treatment (Estimate = 1.94 ± 1.07, *z* = 1.82, *p* = 0.0688; Table 2). However, pairwise post hoc tests did not indicate significant deviations in bee capture across compounds.

**Table 2:** Fixed effects results from negative binomial mixed models predicting squash bee capture at blue vane traps baited with EAG-active compounds accounting for temporal variation across days. The table shows the estimates for each compound tested, with associated standard errors, z-values, and p-values. The estimated random intercept variation for day was 3.849 ± 1.962. The high model uncertainty for indole and linalool reflect that traps baited with both of these compounds did not capture any bees

Compound	Estimate	Std. Error	*z* value	*p* value
Intercept	−3.602	1.460	−2.468	0.0136 ^*^
1,4-dimethoxybenzene	−1.944	1.068	1.820	0.0688
β-ionone	−1.097	1.154	0.951	0.342
dihydro-β-ionone	−0.069	1.224	0.564	0.573
(*E*)-nerolidol	−1.608	1.095	1.469	0.142
indole	−11.711	34.790	−0.034	0.973
linalool	−11.711	34.790	−0.034	0.973
methyl salicylate	−0.691	1.224	0.565	0.572

Significance codes: ^**^p < 0.01; ^*^p < 0.05; *p* < 0.1

### Emissions of EAG-active Compounds Predict Squash Bee Visitation and Nectaring

We identified significant negative correlations between emissions of 1,4-dimethoxybenzene and linalool (t = −82.911, df = 3430, *p* < 0.001, Pearson’s correlation = −0.817; VIF = 4.022) as well as 1,4-dimethoxybenzene and methyl salicylate (t = −51.893, df = 3430, *p* < 0.001, Pearson’s correlation = −0.663; VIF = 6.028). Further, we found significant positive correlations between emissions of linalool and methyl salicylate (t = 99.372, df = 3430, *p* < 0.001, Pearson’s correlation = 0.862; VIF = 3.872).

**Fig. 3:**
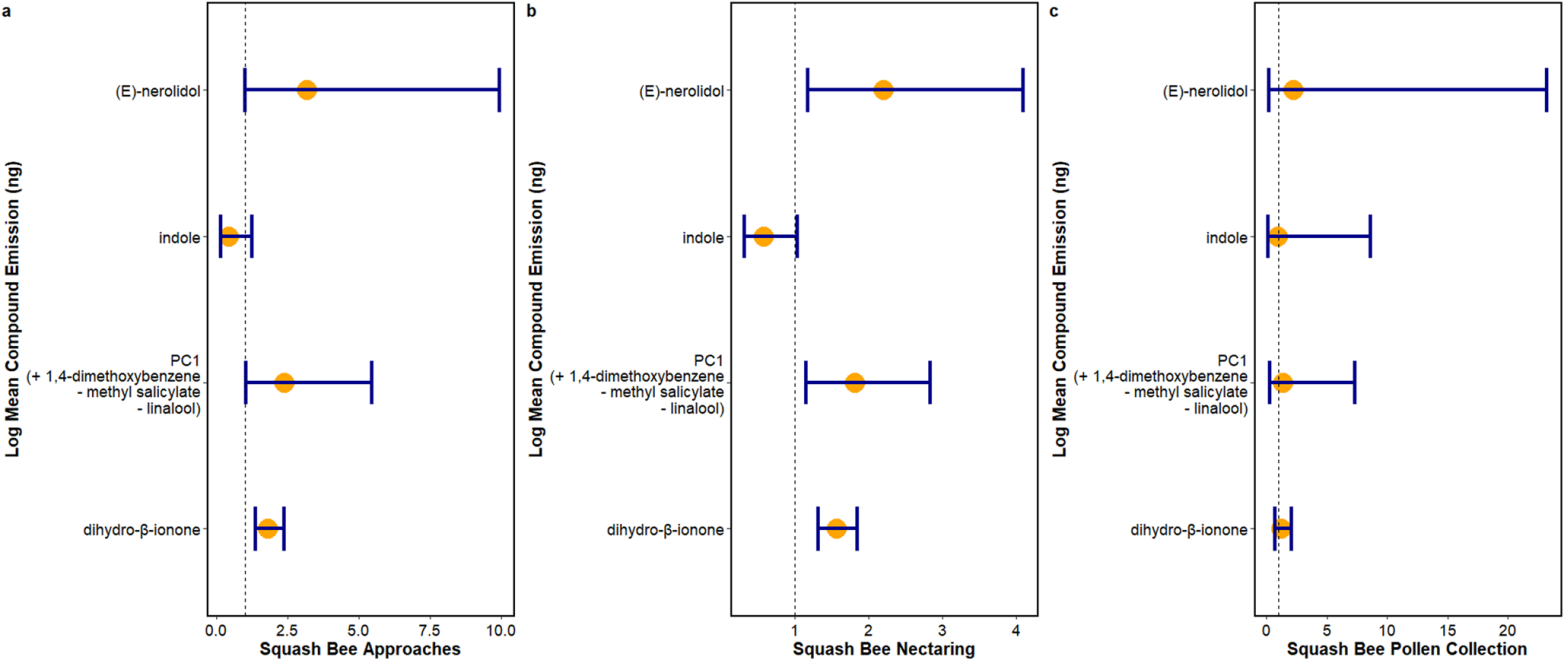
Squash bee behaviour related to mean compound emissions per plant species. Incidence rate ratios of squash bee behavioral events (**a**) approaches, (**b**) nectaring, and (**c**) pollen collection with mean emissions of EAG-active compounds emitted per plant species sampled. PC1 indicates the first eigenvector of a principle component analysis representing autocorrelated emissions of the compounds 1,4-dimethoxybenzene, linalool, and methyl salicylate. We found significant negative correlations between emissions of 1,4-dimethoxybenzene with linalool and methyl salicylate and significant positive correlations between emissions of linalool and methyl salicylate. The dots show the exponentiated coefficients (IRRs) for each compound, indicating the multiplicative change in the expected behavior for a one-unit increase in the compound emissions and the bars represent 95% confidence intervals to indicate. The dotted red line represents the reference IRR value of 1

Using negative binomial linear mixed models, we found that squash bee approaches significantly varied across species (Est: 5.828, Std. error: 1.048, Z = 5.559, *p* < 0.001); *C. foetidissima* plants showed higher numbers of approaches than any other species considered (pairwise *p* < 0.01) and *C. maxima* ssp *maxima* had significantly higher approaches than *C. pepo* ssp. *ovifera* var. *ovifera* (*p* = 0.0232). Further, we found that squash bee approaches significantly varied with emissions of EAG-active compounds (Est: 1.876, Std. error: 0.0936, Z = 20.035, *p* < 0.001; Fig. 3a). Specifically, bee approaches significantly increased with increases in (*E)*-nerolidol emissions (Est: 1.151, Std. error: 0.584, Z = 1.969, *p* = 0.0489), dihydro-β-ionone emissions (Est: 0.586, Std. error: 0.142, Z = 4.116, *p* < 0.001), and the eigenvector representing increased 1,4-dimethoxybenzene and reduced linalool and methyl salicylate emissions (Est: 0.876, Std. error: 0.422, Z = 2.053, *p* = 0.0400).

We obtained similar results for squash bee nectaring events, which also significantly varied across squash species considered for this study (Est: 3.422, Std. error: 0.557, Z = 6.148, *p* < 0.001). Pairwise post hoc tests revealed that *C. foetidissima* plants experienced higher numbers of nectaring events than any other species considered (pairwise *p* < 0.01) and *C. maxima* ssp *maxima* had significantly higher approaches than *C. pepo* ssp. *ovifera* var. *ovifera* (*p* = 0.00798). Again, the number of observed squash bee nectaring events significantly varied with emissions of EAG-active compounds (Est: 1.448, Std. error: 0.050, Z = 28.765, *p* < 0.001; Fig. 3b). Nectaring events significantly increased with increases in (*E)*-nerolidol emissions (Est: 0.788, Std. error: 0.318, Z = 2.481, *p* = 0.0131), dihydro-β-ionone emissions (Est: 0.448, Std. error: 0.0854, Z = 5.251, *p* < 0.001), and the eigenvector representing increased 1,4-dimethoxybenzene and reduced linalool and methyl salicylate emissions (Est: 0.596, Std. error: 0.228, Z = 2.611, *p* = 0.00902).

Our models indicated that while the number of pollen collection events significantly varied across species (Est: 0.104, Std. error: 0.0340, Z = 3.048, *p* = 0.00231), pairwise post hoc tests did not recover significant pairwise differences across species. Similarly, the number of squash bee pollen collection events also varied significantly with EAG-active compound emissions (Est: −2.587, Std. error: 0.177, Z = −14.624, *p* < 0.001; Fig. 3c). However, we found no significant pairwise associations between pollen collection events and the emissions of any compounds tested.

## Discussion

In this study, we identified floral volatile compounds within wild and domesticated *Cucurbita* species that are antennally active and associated with squash bee visitation to *Cucurbita* flowers using a combination of volatile headspace sampling, electrophysiology, and behavioral assays. We show that domestication is associated with shifts in floral volatile profiles primarily driven by losses of floral volatile compounds and the retention of 1,4-dimethoxybenzene. We observed ten electrophysiologically-active floral compounds across volatile mixtures of all five species and tentatively confirmed the identity of seven compounds. Trap-based assays showed that 1,4-dimethoxybenzene was sufficient to attract bees in isolation. Pollinator visitation experiments indicate that three compounds: 1,4-dimethoxybenzene, (*E*)-nerolidol, and dihydro-β-ionone, were positively associated with increased bee approaches and nectaring, while linalool and methyl salicylate showed negative associations with these behaviors. Interestingly, we found that 1,4-dimethoxybenzene is only present in species occurring in the mesic clade and it is the only compound that persists in the heavily domesticated *C. pepo* ssp. *ovifera* var. *ovifera*, suggesting that this volatile is likely functionally important for domesticated plants. (*E*)-nerolidol showed an opposite phylogenetic pattern, where it is present in all species apart from *C. pepo* ssp. *ovifera* var. *ovifera* and is particularly abundant in the wild species *C. foetidissima* and *C. maxima* ssp. *andreana*. Dihydro-β-ionone is emitted by both *C. pepo* ssp. *ovifera* var. *ovifera* and *C. foetidissima*. Taken together, our results demonstrate that domestication alters signals used by these specialist pollinators to locate floral resources and provide new insights into plant-pollinator interactions in agroecosystems.

The compound 1,4-dimethoxybenzene is a methoxylated aromatic volatile compound that is produced across plant genera and is a common attractant for insects to flowers (Burger et al. 2013; Jürgens et al. 2014). This compound was present in all the species sampled within the mesic clade of *Cucurbita* and absent in the wild xeric species *C. foetidissima.* In the most domesticated species in our study, *C. pepo* ssp. *ovifera* var. *ovifera* (Castellanos-Morales et al. 2018; Smith 2006)), 1,4-dimethoxybenzene accounts for the majority of floral volatile blends (> 90%). This compound, in isolation, was also the strongest attractant for squash bees in the blue vane trap field assays in this study. However, our analyses of pollinator visitation indicated that while higher 1,4-dimethoxybenzene emissions were associated with increased approaches and nectaring by squash bees, they were not associated with pollen collecting behavior. Our results mirror those of previous studies that demonstrate the importance of this compound to attract squash bees and specialist herbivores to cucurbit flowers (Barman et al. 2023; Theis and Adler 2012). Indeed, 1,4-dimethoxybenzene has been identified as an olfactory cue for both specialist and generalist bee pollinators across plant species. For instance, in *Salix caprea*, the emission of this compound is associated with visitation of both the specialist *A. vaga* (Dötterl et al. 2005) and the generalist *A. mellifera* (Jürgens et al. 2014).

Interestingly, we found that emissions of 1,4-dimethoxybenzene were negatively correlated with those of the compounds linalool and methyl salicylate. This finding might reflect underlying tradeoffs between biosynthetic pathways involved in the production of these compounds, as all three are produced via distinct, intersecting metabolic pathways (Lv et al. 2024). 1,4-dimethoxybenzene and methyl salicylate are benzenoids, produced via the methylation of 4-methoxyphenol and salicylic acid (Hoepflinger et al. 2024), respectively, while linalool is a monoterpene compound synthesized through the action of linalool synthases within the MEP pathway (Raguso 2016). The production of these compounds may be constrained by a limited pool of available substrates, cofactors, and regulatory elements, particularly in the case of 1,4-dimethoxybenzene and methyl salicylate, which are both produced via the action of methyltransferase enzymes (Dudareva et al. 2013). Future transcriptomic studies focusing on the compounds highlighted in this study might provide mechanistic evidence of these hypothesized tradeoffs between compound production.

There is mounting evidence that the activity of both herbivores and pollinators together shape the volatile blends emitted by flowers. As floral volatiles are byproducts of plant metabolic and defense pathways, volatile profiles often change in response to ecological challenges plants face. Insects foraging on plants are quick to form associations between floral signals and reward quality (Sasidharan et al. 2023; Schiestl 2015). As florivores and pollinators often use the same compounds to detect specific plants, they often exert opposing selective pressures on compound emission (Raguso 2008). This has been previously demonstrated in the *Cucurbita* system; using *C. pepo* ssp. *ovifera* var. *texana,* Theis and Adler (2012) showed that artificially increased 1,4-dimethoxybenzene emissions were attractive to pollinators in the absence of the florivore *A. vittatum* but repellant when this species was abundant. Our work demonstrates that, despite the previously established attractiveness of 1,4-dimethoxybenzene to herbivorous species, the retention of this compound in domesticated plants might signal its importance to pollinator visitation. We also generated putative identifications for several plant defense and herbivore repellent compounds that are associated with increased pollinator visitation to squash plants. Increased herbivory has been shown to deter pollinator activity in *Cucurbita* and other insect-pollinated plants (Leach and Kaplan 2025) and thus, the presence of plant compounds repellant to herbivores might signal their absence and attract foraging bees (Kessler et al. 2019). For example, Kessler et al. (2011) showed that simulating herbivory-induced floral volatile changes by adding methyl jasmonate to wild tomato flowers increased the visitation of native pollinators. Several previous studies have explored induced defense mechanisms and their effects on floral volatiles in *Cucurbita* and our understanding of the effects of defense compounds on pollinator species is expanding (Barrett et al. 2021; Hinshaw et al. 2024).

The domestication and widespread cultivation of their host plant species have posed novel ecological challenges for squash bees. Our study demonstrates that domesticated squash flowers have significantly different chemical profiles from the wild flowers that are the hypothesized ancestral host of *X. pruinosa*, despite unequal variances. Similar shifts in floral volatile blends post-domestication have been shown for other cultivated plant groups such as roses (Feng et al. 2022) and cotton (Hagenbucher et al. 2016). The dominance of 1,4-dimethoxybenzene within domesticated squash species and its absence in the wild ancestral host species coupled with the dominance of (*E*)-nerolidol in wild *C. foetidissima* might indicate shifts in signalling cues used by bees within domesticated squash species. These shifts in volatile production might be due to domesticated plants being typically more susceptible to herbivores due to attenuated defense traits and inbreeding depression (Whitehead et al. 2017). This has been demonstrated in *C. argyrosperma* ssp. *argyrosperma*, where domesticated plants produce much lower levels of defensive compounds than wild plants (Jaccard et al. 2023). Crop plants are often associated with dramatically different insect communities than domesticated plants, including pest species (Chen et al. 2018). The preferences of newly acquired herbivore and pollinator species might exert opposing selective forces on floral traits, causing rapid phenotypic change (Glasser et al. 2023).

Other floral visitors that might influence floral volatile blends in *Cucurbita* species are generalist pollinator species, such as *A. mellifera* and *Bombus* spp., that visit squash flowers to collect the abundant nectar (Shuler et al. 2005). Bumble bees, in particular, have been shown to be effective pollinators of squash (Stoner 2020). The expansion of cultivated squash species has resulted in a recent large range expansion for these plants across North America, a process that resulted in altered ecologies and interaction with new pollinator species (Pope et al. 2023). Generalist bee species have been shown to use common floral volatiles that are major components of volatile blends, such as 1,4-dimethoxybenzene, while foraging (Kantsa et al. 2019). In contrast, specialists generally rely on compounds that are minor components of blends (Milet-Pinheiro et al. 2013). However, our results show that specialist bees use volatile compounds that are likely also detected by generalists to find flowers, potentially indicating that generalist preferences might also be exerting stabilizing selection on the persistence of these volatile blends. Future work will test this hypothesis by conducting GC-EAG and behavioral assays on generalist pollinators of squash.

It is important to note that the results of our study should be interpreted in the light of several caveats, the most important of which concerns the varied ecological conditions present across the range of domesticated and wild squash. *Cucurbita foetidissima* occurs within xeric habitats within the Southwestern US and Northern Mexico and thus has distinct phenology and grows in different environmental conditions compared to the other squash species considered. Future work might sample other xerophytic *Cucurbita* species to understand whether these abiotic conditions might also drive floral volatile profiles. Further, reliance on specific sensory cues varies across an insect’s lifetime: naive bees may be more dependent on olfactory cues than experienced bees (Milet-Pinheiro et al. 2013; Rusch et al. 2016). We were not able to determine the age of the bees we used in our trials and thus cannot differentiate between naive and experienced bees within our data. A final caveat is that we did not conduct behavioral tests to compare the effects of volatiles within blends, an approach that may allow us to determine the behavioral valence of individual compounds both in blends and in isolation. Future work should use the compounds described within this study as a starting point for multimodal explorations of plant-pollinator interactions in this system.

This study advances our understanding of the ecology and evolution of wild and domesticated *Cucurbita*, and provides new insights into the chemical ecology of plant-pollinator interactions in agroecosystems. There are several other crops that are visited by specialist pollinators, including blueberries, tomatoes, and sunflowers. Future studies might focus on understanding the chemical ecology of plant-pollinator interactions within these crops and the ways in which domestication might change plant traits and communication with pollinators. Broadening our understanding of the chemical signals used by pollinators while visiting these crop plants may lead to management and breeding strategies to enhance crop yields via improved pollination for these species (Jones and Rader 2022).

## Supplementary Information

Below is the link to the electronic supplementary material.

Supplementary file1 (XLSX 8 KB)

Supplementary file2 (XLSX 54 KB)

Supplementary file3 (XLSX 34 KB)

Supplementary file4 (XLSX 45 KB)

Supplementary file5 (XLSX 50 KB)

Supplementary file6 (XLSX 50 KB)

Supplementary file7 (XLSX 230 KB)

## Data Availability

Data is provided within the attached supplementary information files. Scripts used to coduct analysis will be made publicly available at https://github.com/Avehi upo acceptance of this manuscript.
